# Passive Dendrites Enable Single Neurons to Compute Linearly Non-separable Functions

**DOI:** 10.1371/journal.pcbi.1002867

**Published:** 2013-02-28

**Authors:** Romain Daniel Cazé, Mark Humphries, Boris Gutkin

**Affiliations:** 1 Group for Neural Theory, INSERM U960, Ecole Normale Superieure, Paris, France; 2 Paris 7 Diderot, Paris, France; 3 Faculty of Life Sciences, University of Manchester, Manchester, United Kingdom; Indiana University, United States of America

## Abstract

Local supra-linear summation of excitatory inputs occurring in pyramidal cell dendrites, the so-called dendritic spikes, results in independent spiking dendritic sub-units, which turn pyramidal neurons into two-layer neural networks capable of computing linearly non-separable functions, such as the exclusive OR. Other neuron classes, such as interneurons, may possess only a few independent dendritic sub-units, or only passive dendrites where input summation is purely sub-linear, and where dendritic sub-units are only saturating. To determine if such neurons can also compute linearly non-separable functions, we enumerate, for a given parameter range, the Boolean functions implementable by a binary neuron model with a linear sub-unit and either a single spiking or a saturating dendritic sub-unit. We then analytically generalize these numerical results to an arbitrary number of non-linear sub-units. First, we show that a single non-linear dendritic sub-unit, in addition to the somatic non-linearity, is sufficient to compute linearly non-separable functions. Second, we analytically prove that, with a sufficient number of saturating dendritic sub-units, a neuron can compute all functions computable with purely excitatory inputs. Third, we show that these linearly non-separable functions can be implemented with at least two strategies: one where a dendritic sub-unit is sufficient to trigger a somatic spike; another where somatic spiking requires the cooperation of multiple dendritic sub-units. We formally prove that implementing the latter architecture is possible with both types of dendritic sub-units whereas the former is only possible with spiking dendrites. Finally, we show how linearly non-separable functions can be computed by a generic two-compartment biophysical model and a realistic neuron model of the cerebellar stellate cell interneuron. Taken together our results demonstrate that passive dendrites are sufficient to enable neurons to compute linearly non-separable functions.

## Author Summary

Classical views on single neuron computation treat dendrites as mere collectors of inputs, that is forwarded to the soma for linear summation and causes a spike output if it is sufficiently large. Such a single neuron model can only compute linearly separable input-output functions, representing a small fraction of all possible functions. Recent experimental findings show that in certain pyramidal cells excitatory inputs can be supra-linearly integrated within a dendritic branch, turning this branch into a spiking dendritic sub-unit. Neurons containing many of these dendritic sub-units can compute both linearly separable and linearly non-separable functions. Nevertheless, other neuron types have dendrites which do not spike because the required voltage gated channels are absent. However, these dendrites sub-linearly sum excitatory inputs turning branches into saturating sub-units. We wanted to test if this last type of non-linear summation is sufficient for a single neuron to compute linearly non-separable functions. Using a combination of Boolean algebra and biophysical modeling, we show that a neuron with a single non-linear dendritic sub-unit whether spiking or saturating is able to compute linearly non-separable functions. Thus, in principle, any neuron with a dendritic tree, even passive, can compute linearly non-separable functions.

## Introduction

Seminal neuron models, like the McCulloch & Pitts unit [1] or point neurons (see [2] for an overview), assume that synaptic integration is linear. Despite being pervasive mental models of single neuron computation, and frequently used in network models, the linearity assumption has long been known to be false. Measurements using evoked excitatory post-synaptic potentials (EPSPs) have shown that the summation of excitatory inputs can be supra-linear or sub-linear [3], [4], [5], [6], [7], [8], [9], [10], [11], [12], and can summate in quasi-independent regions of dendrite [13].

**Figure 1:**
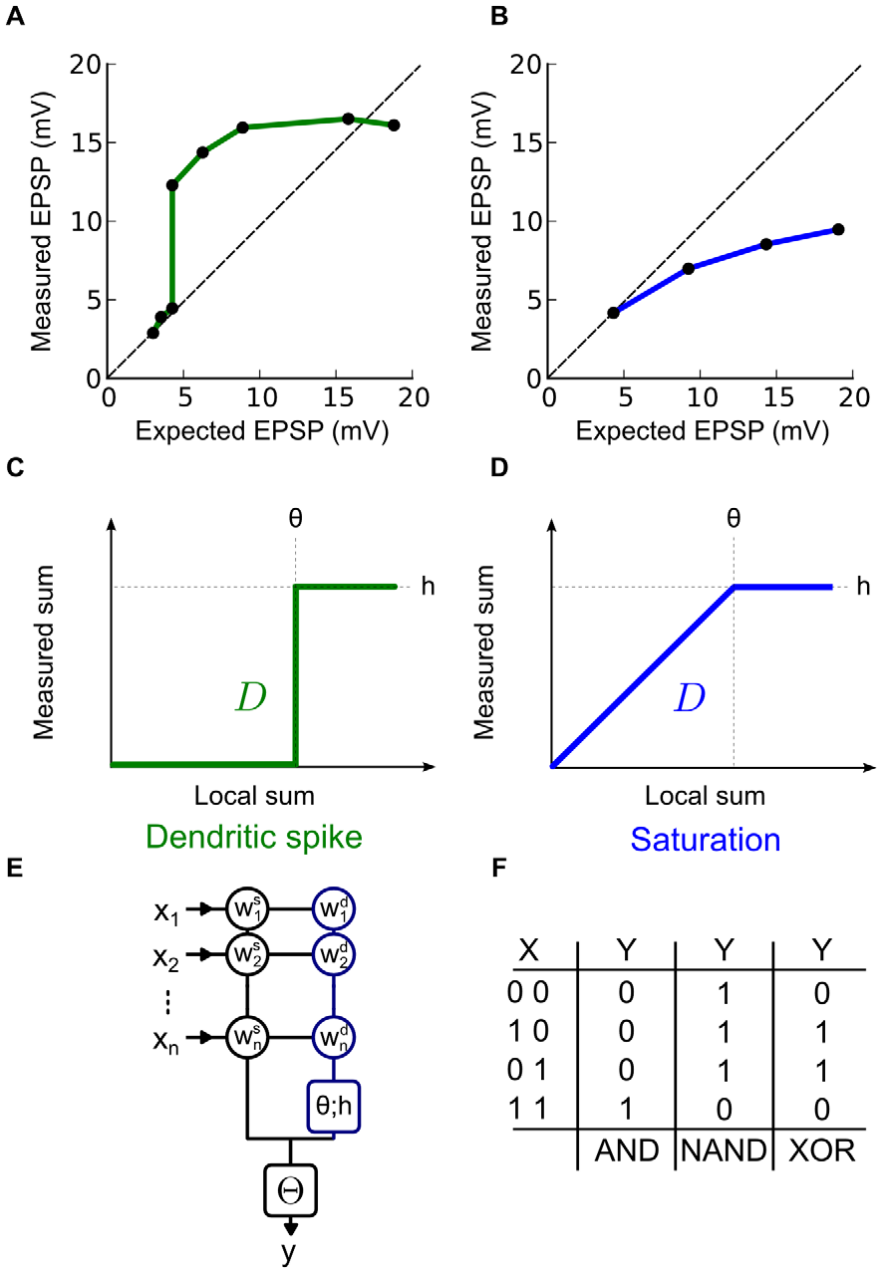
Two types of local dendritic non-linearities. (A–B) The x-axis (Expected EPSP) is the arithmetic sum of two EPSPs induced by two distinct stimulations and y-axis (Measured EPSP) is the measured EPSP when the stimulations are made simultaneously. (A) Observations made on pyramidal neurons (redrawn from [13]). Summation is supra-linear and sub-linear due to the occurrence of a dendritic spike. (B) Â Observations made on cerebellar interneurons (redrawn from [10]). In this case summation is purely sub-linear due to a saturation caused by a reduced driving force. (C) Â The activation function modeling the dendritic spike type non-linear summation: both supra-linear and sub-linear on 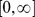. (D) Â The activation function modeling the saturation type non-linear summation: strictly sub-linear on 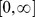. (E) Structure and parameters of the neuron model: 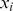 and 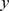 are binary variables describing pre and post-synaptic neuronal activity; in circles are two independent sets of non-negative integer-valued synaptic weights respectively for the linear (black) and the non-linear integration (blue) sub-units; in the blue square, 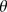 and 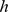 are the non-negative integer-valued threshold and height that parameterize the dendritic activation function 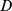; in the black square 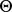 is a positive integer-valued threshold determining post-synaptic firing. (F) Â Truth tables of three Boolean functions for 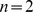 inputs: AND, NAND, and XOR. The first column gives the possible values of the input vector 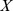; the other three columns give the binary outputs 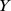 in response to each 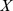 for the three functions considered.

Supra-linear summation, the dendritic spikes, has been described for a variety of active dendritic mechanisms. For this type of local summation the measured EPSP peak is first above then below the expected arithmetic sum of EPSPs as shown on Figure 1A. Synapse-driven membrane potential depolarization can open 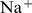 [3], [4], 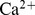 [5], [6], or NMDA receptor [6], [7], [8], [9] channels sufficiently to amplify the initial depolarization, and evoke a dendritic spike.

Contrary to the supra-linear summation of dendritic spikes, a saturating sub-linear summation can arise from passive properties of the dendrite [10], [11], [12]. For this type of local summation the measured EPSP peak is always below the expected arithmetic sum of all EPSPs as shown on Figure 1B. Rall's theoretical work [14], [15], subsequently confirmed experimentally [12], showed that passive sub-linear summation of overlapping inputs is a straightforward consequence of the classic model 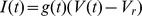 for conductance-driven current injection into the membrane (where 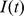, 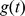, and 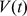 are respectively the time varying current, the synaptic conductance, and the membrane voltage, and where 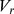 is the equilibrium voltage of the channel).

Dendritic spikes inevitably alter the potential range of single neuron computation. Prior theoretical studies found that dendrites could be divided up into multiple, independent sub-units of integration [16], [17], [18], [19], [20] with sigmoidal or Heaviside activation functions (as shown on Figure 1C). They argued that these dendritic spikes turn synaptic integration into a two stage process: first, synaptic inputs are summed in independent sub-units; second, the output of these sub-units is linearly summed at the soma. Such a two-stage architecture makes the neuron computationally equivalent to a two-layer artificial neural network, greatly expanding a neuron's computational capacities. It has been shown that spiking dendritic sub-units can enhance the feature storage capacity [18], the generalization capacity [17], [21], the computation of binocular disparity [22], the direction selectivity [23], [24], the creation of multiple place fields [25] or the computation of object-feature binding problems [26]. These enhancements may be explained by the ability of a neuron with a sufficient number of spiking dendritic sub-units to compute linearly non-separable functions whereas seminal neuron models like McCulloch & Pitts cannot [27].

These prior studies made two assumptions that may not generalize to all neurons. Firstly, they supposed that the number of independent dendritic sub-units is potentially large; however, for different dendritic morphologies this number may be greatly reduced due to electrotonic coupling or compactness [28], [29], [30]. Secondly, dendritic spikes may not be present in all neuron types, because they lack the specific voltage-gated channels or because the active channel types act to balance each other [10], [11], [31], [32]. Consequently these neuron types could only support a saturating form of non-linear integration. The cerebellar stellate cell is an interesting example because it contradicts both assumptions: it is electrically compact, resulting in a modest number of independent dendritic non-linear sub-units, perhaps on the order of 10 sub-units, as has also been estimated for retinal ganglion cells [33]; and its dendrites are passive, with linear integration of inputs in the peri-somatic region and strictly sub-linear integration in the distal dendritic region [10].

If non-linear computation by dendrites were possible for small numbers of sub-units and for passive dendrites, then this would show that enabling linearly non-separable computation by single neurons is, in principle, a general property of dendrites. We thus set out to answer three key questions: (1) whether a single non-linear dendritic sub-unit is sufficient to enable a neuron to compute linearly non-separable functions, so that multiple sub-units are not a necessary requirement; (2) whether saturating dendritic sub-units, and not just a spiking dendritic sub-units, are sufficient to enable a neuron to compute linearly non-separable functions; and (3) if so, whether the saturating and spiking non-linearities increase computational capacity in the same way.

To answer these questions, we have used a binary neuron model that accounts for non-linear dendritic integration, using either spiking (Figure 1C) or saturating (Figure 1D) activation functions. Using a binary model (Figure 1E) allowed us to study the quantitative increase and qualitative changes in computational capacity using Boolean algebra [34]. A Boolean function is defined by a set of 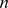 input variables, each taking the value 0 or 1, and a target output value of 0 or 1 for each 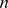-dimensional vector that can be made by all possible combinations of values of the input variables (see Material and Methods Boolean Algebra for formal definition). Figure 1F illustrates three well-known examples of Boolean functions, each a function of 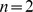 input variables: AND, NAND, and XOR. The set of Boolean functions computable by a binary neuron model provides a lower bound on the realm of potentially computable algebraic functions by a neuron. Thus, specifying capacity in terms of Boolean functions lets us list the boundaries on a neuron's accessible set of all computable functions.

Using this model, we proceeded on two fronts: first, we used numerical analysis to test if and how much an additional non-linear dendritic sub-unit enables a neuron to compute linearly non-separable functions; second, we used formal analytical proofs to show that the numerical results generalise to an arbitrary number of non-linear sub-units. We found numerically that adding a single non-linear dendritic sub-unit, either a spiking or a saturating unit, allows the neuron to compute **some** positive linearly non-separable Boolean functions. Analytically, we showed that provided a sufficient number of either spiking or saturating dendritic sub-units a neuron is capable of computing all positive linearly non-separable Boolean function.

Second, our numerical analysis showed that a neuron could compute a function using two distinct implementation strategies: a local strategy where each dendritic sub-unit can trigger a somatic spike, implying that the maximal responses of a dendritic sub-unit always correspond to a somatic spike; and a global strategy where a somatic spike requires the activation of multiple dendritic sub-units, implying that the maximal response of a dendritic sub-unit may not correspond to a somatic spike. This last result may explain why neurons in layer 2/3 of the visual cortex can stay silent when a calcium response from a dendritic sub-unit is maximal [35]. Analytically, we prove that a neuron with spiking dendritic sub-units can use both strategies to compute a function, whereas a neuron with saturating dendritic sub-units can use only a global strategy to compute a function.

Finally, we show how examples of linearly non-separable functions can be implemented in a reduced, generic biophysical model with either a saturating or a spiking dendrites. Moreover, we show that with electrically compact and passive dendrites, a realistic biophysical model of the cerebellar stellate cell can compute a linearly non-separable function. In conclusion, our study thus extends prior work [16], [19], [20] to show that even a compact neuron with passive dendrites can compute linearly non-separable functions.

### Modelling two types of local and non-linear summation

We present in this section the binary neuron models we used to address our questions with a numerical and a formal analysis. We considered two types of dendritic non-linearities modeled by two families of non-linear activation functions 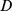, (Figure 1B–C and definition in Materials and Methods spiking and saturating dendritic activation functions). The first family, 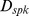, modeled dendritic spikes as observed in [6], [13], [4]. The second family, 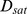, modeled dendritic saturation as observed in [12], [10], [11]. Both are parameterized by two non-negative parameters for threshold 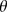 and maximum output 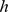.

For our numerical analysis, based on large parameter searches, we added the output of either of these activation functions 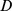 to a strictly linear sub-unit integrating the same inputs:
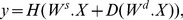
(1)where 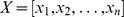 is a binary input vector of length 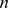, 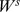 and 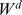 are non-negative integer-valued weight vectors, and the somatic activation function 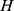 gives 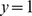 if the result of synaptic integration is above 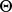 and 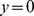 otherwise. (Note that if 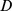 is a linear function (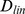) then the previous equation can be rewritten as a single linear weighted sum corresponding to the seminal linear neuron model known as the McCulloch & Pitts unit [1]). Poirazi et al [18] have already established that a single non-linear dendritic sub-unit on its own is not sufficient to increase a neuron model's computation capacity. We thus added a non-linear dendritic sub-unit to the somatic non-linearity precisely to assess the impact of adding either a spiking dendritic non-linearity (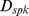) or a saturating dendritic non-linearity (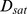) on single neuron computation. As a corollary, this model includes neuron classes that have a peri-somatic and a distal dendritic region of integration, like cerebellar stellate cell interneurons [10] and layer 2/3 pyramidal neurons [36].

For our formal analysis, which are the three Propositions presented in the Results, we used the generic two-stage neuron model with 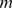 dendritic sub-units, analogous to [18]:
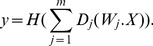
(2)For both our numerical and formal analysis, the neuron input-output mapping is defined as a Boolean function, and each parameter set produces a unique Boolean function 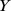. Here we focused on the effect of non-linear EPSP summation, and thus used only non-negative weight vectors. Consequently, an increase in input 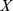 could only increase (or not change) the output 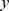, never decrease it; therefore we were studying the neuron's ability to compute positive Boolean functions (see Material and Methods Boolean Algebra for formal definition and Lemma 1 for proof).

In terms of neuron physiology, these binary models are quite general. One can interpret the binary input vector across multiple scales, from the pattern of active and inactive individual pre-synaptic neurons up to the set of active and inactive pre-synaptic cell assemblies afferent to the neuron. In this perspective, the weight represents the peak EPSP magnitude produced when a pre-synaptic neuron or a pre-synaptic cell assembly is active. Similarly the Boolean output of 1 could represent a single spike, a burst of spikes, or a change in rate – whatever it is that is read out by the downstream neurons. For instance, in our biophysical model, a single binary variable 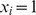 corresponds to the synchronous activity of a 100 pre-synaptic neurons, and will show that binary models can lead to informative results in situations where the actual number of pre-synaptic input neurons is in the range consistent with existing data.

## Results

### The computation capacity enabled by spiking or saturating dendrites

We first sought answers to two questions: (1) whether a single non-linear dendritic sub-unit is sufficient to enable a neuron to compute linearly non-separable functions, so that multiple sub-units are not a necessary requirement; (2) whether saturating dendritic sub-units, instead of spiking dendritic sub-units, are sufficient to enable a neuron to compute linearly non-separable functions. For a given size 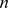 of the input vector 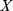, we numerically enumerated the sets of positive Boolean functions computable by the different models (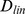, 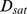, or 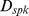), by searching through their free parameters: the dendritic sub-unit activation function parameters 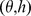, the weight vectors 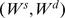, and the neuron output threshold 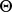. For each set of parameter values, we computed the corresponding Boolean function for that neuron model. This numerical analysis enabled us to determine the computational capacity for each neuron model (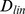, 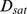, and 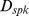) as the number of Boolean functions these models can compute.

**Figure 2:**
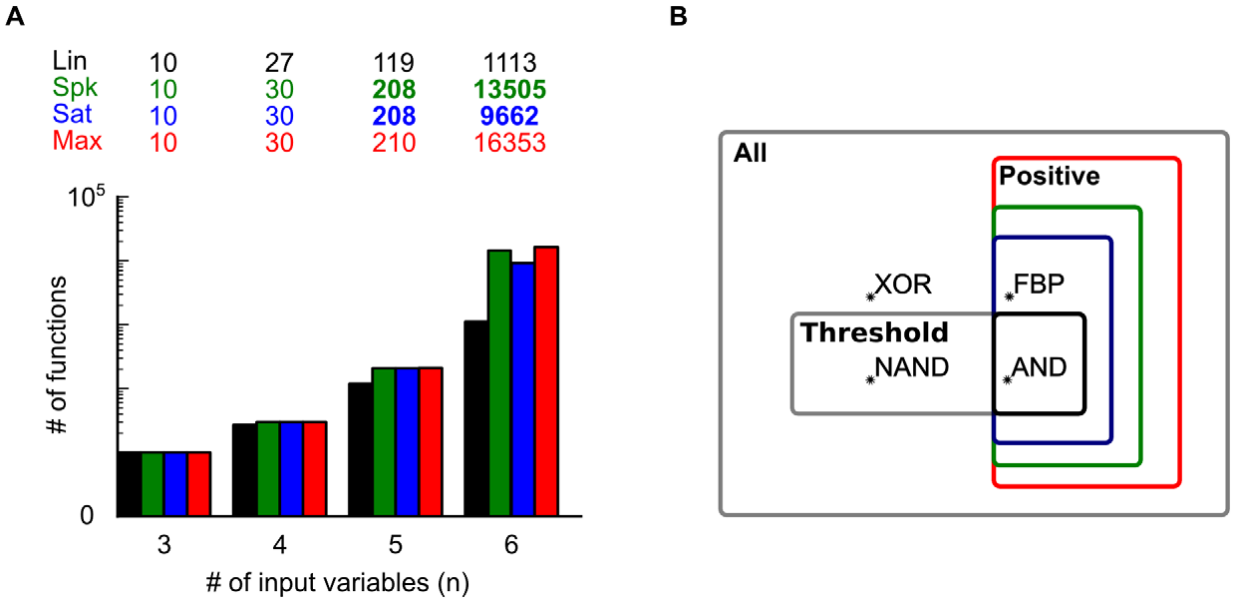
A dendritic non-linearity enables the computation of linearly non-separable Boolean functions. (A) Number of computable representative positive Boolean functions depending on the number of input variables 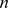 and on the type of synaptic integration: purely linear (lin∶black), linear with a spiking dendritic sub-unit (spk∶green), linear with saturating dendritic sub-unit (sat∶blue). In red is the maximal number of positive representative functions computable for a given 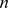, this number is taken from [37] as the number of functions in condition lin (black). Upper panel: number of computable functions (in bold are lower bounds); lower panel: summary bar charts on logarithmic scale. (B) Venn diagram for the sets of Boolean functions for 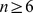. The set border color depends on the type of integration, as per panel A (relative size of sets not to scale). Stars are examples of Boolean functions within each set.

To determine the computational capacity, we counted only the computable representative Boolean functions (see Materials and Methods Boolean Algebra for formal definition). Moreover, we controlled the parameter searches using two analytically known sizes of Boolean function sets: first, the size of the set of all representative positive Boolean functions [34], [37], known for a number of binary variables 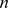 up to 6; second, within this set of functions, the number of linearly separable representative Boolean functions [37]. This last number corresponded to the exact computational capacity for the purely linear model (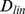). Therefore by comparing to these two known sizes we could see if the model including a dendritic non-linearity (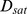 or 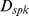) enabled computation of linearly non-separable functions and, if so, what proportion of those functions could be accessed. The relationship between these sets of functions and the set that can be accessed by a model including a non-linear dendritic sub-unit is illustrated schematically in Figure 2B.

#### A spiking dendritic nonlinearity enables a neuron to compute linearly non-separable functions

Figure 2A shows that the addition of a single spiking sub-unit (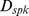) is sufficient to increase the computational capacity of a neuron model, by enabling the neuron to compute linearly non-separable functions. For 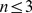 input variables, the computation capacity was the same with or without a dendritic spiking sub-unit. For 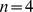, the addition of the spiking sub-unit enabled the computation of 3 new Boolean functions. For 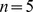, the advantage was 89 new Boolean functions. Finally, for 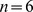, the spiking sub-unit enabled the computation of over 9000 new Boolean functions. For all tested 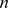, a spiking dendritic sub-unit enabled a neuron to compute all linearly separable Boolean functions computable by a neuron with purely linear integration. These numerical results show that a single spiking dendritic sub-unit is sufficient for a two-stage neuron to compute a substantial fraction of linearly non-separable and positive Boolean functions. Interestingly, these numerical results also show that the addition of a single spiking dendritic sub-unit does not allow a neuron to compute *all* positive functions, as it is already happening for 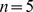.

Analytically, we can easily show that such a neuron, equipped with a sufficient number of spiking sub-units, can compute every linearly non-separable positive Boolean function. This result is equivalent to the well-known proof that a two-layer neural network with supra-linear activation functions can compute all Boolean functions (see [38] Theorem 13.9). The following proposition restricts this Theorem to positive Boolean functions and it explicitly describes the method to implement a function. We briefly state the proof here, which follows from the Lemmas and definitions given in Materials and Methods.

*Proposition 1*. A two stage neuron (Eq. 2) with non-negative synaptic weights and a sufficient number of dendritic units with spiking activation functions can implement only and all positive Boolean functions based on their positive complete disjunctive normal form (DNF)

*Proof*. A two stage neuron can only compute positive Boolean functions (Lemma 1). All positive Boolean functions can be expressed as a positive complete DNF; because a spiking dendritic unit has a supra-linear activation function it can implement any of the possible terms in that DNF (Lemma 2). Therefore, a two stage neuron model without inhibition can implement only and all positive Boolean functions with as many dendritic units as there are terms in the functions' positive complete DNF.

While this proof is general, it does not necessarily imply that a real neuron can compute a linearly non-separable function with a small finite number of spiking sub-units. However, our numerical results show that even a single dendritic spiking sub-unit is sufficient to access the space of linearly non-separable functions.

#### A saturating dendritic nonlinearity also enables a neuron to compute linearly non-separable functions

Figure 2A shows that the addition of a single saturating sub-unit (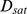) is also sufficient to increase computational capacity by enabling a neuron to compute linearly non-separable functions. Similar to the spiking sub-unit, a saturating sub-unit enables the computation of the 3 linearly non-separable functions for 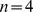. Moreover, our numerical results showed that the addition of the single saturating sub-unit (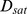) allowed the neuron to compute up to 9000 more functions than the purely linear neuron model (for 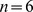). The numerical analysis also showed that, for an identical range of searched parameters at 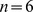, a saturating sub-unit (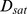) enabled a neuron to compute fewer functions than a spiking sub-unit (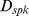), (see Figure 2 and Table 1). We analytically address potential reasons for this difference in the next section. Nonetheless, these numerical results show that a single saturating sub-unit is also sufficient for a two-stage neuron to compute a notable fraction of positive linearly non-separable Boolean functions.

**Table 1:** The integer-valued parameter range used in our parameter searches depending on the neuron model [lin; sat; spk], for at most 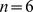 input binary variables.

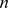	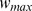	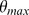	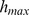	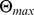
6	[9;4;4]	[-;8;8]	[-;12;12]	[18;20;20]
5	[5;3;3]	[-;3;3]	[-;4;7]	[9;8;12]
4	[3;2;2]	[-;2;2]	[-;2;3]	[5;4;6]

In the first line, numbers give the maximal parameters values used in our searches up to 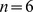. In all lines, the other numbers are the sufficient parameter values, meaning that if one was to launch a parameter search with a value higher than the one given in this table, one would find the same computation capacity as the one presented in Figure 2. For instance for 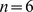, if one wants to implement all positive linearly separable Boolean function with a purely linear neuron, the first line of this table (first element between square brackets) shows that it is sufficient to have integer-valued synaptic weights between 0 and 9, and an integer-valued threshold between 0 and 18; this range is also given in [37]. We built this table using a large parameter search (see Materials and Methods), using this method we computed the Boolean function for more than 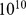 parameter sets.

Analytically, we can also show that a neuron, equipped with a sufficient number of saturating sub-units can compute every linearly non-separable positive Boolean function. We briefly state the proof here, Lemmas and definitions are given in Material and Methods:

*Proposition 2*. A two stage neuron (Eq. 2) with non-negative synaptic weights and a sufficient number of dendritic units with spiking or saturating activation functions can implement only and all positive Boolean functions based on their positive complete conjunctive normal form (CNF)

*Proof*. A two stage neuron can only compute positive Boolean functions (Lemma 1). All positive Boolean functions can be expressed as a positive complete CNF; because a spiking or a saturating dendritic unit has a sub-linear activation function it can implement every possible clause (Lemma 2). Therefore a two stage neuron model without inhibition can implement only and all positive Boolean functions with as many saturating dendritic units as there are clauses in the functions' positive complete CNF.

Consequently, a neuron with sufficient multiple saturating sub-units in its dendrites has, in principle, the same computational capacity as a neuron with spiking sub-units. Moreover, our numerical results extend this to show that, even if a real neuron can only sustain one independent saturating sub-unit, this is sufficient to enhance computation.

### Linearly non-separable functions can be implemented using at least two distinct methods

Having established that saturating dendritic sub-units also enhance the computational capacity of a neuron, we then sought to answer the third question: do the saturating and spiking non-linearities increase computational capacity in the same way? Our aim was to understand how the implementation of linearly non-separable Boolean functions depends on the type of dendritic non-linearity. For concreteness, we numerically assessed the implementation of all three linearly non-separable Boolean functions defined for 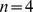 inputs; the functions are presented in Table 2. Interestingly, these three functions map onto known computational problems in neuroscience.

**Table 2:** The partial truth tables for the three linearly non-separable Boolean functions of 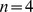 variables.

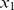	1	1	1	0	0	0
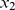	1	0	0	1	1	0
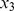	0	1	0	1	0	1
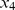	0	0	1	0	1	1
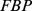	1	0	0	0	0	1
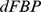	0	1	1	1	1	0
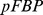	1	1	0	0	0	1

All input vectors inferior (as defined in our definition of positive functions) to the one below give 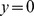; all input vectors superior to the one below give 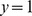.

To help identify these functions we named them in reference to [26]: the Feature Binding Problem (FBP), the dual Feature Binding Problem (dFBP), and the partial Feature Binding Problem (pFBP). In the FBP, the function gives an output only when disjoint sets of inputs are active (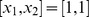 or 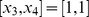) and not when any other mixture is active, and is hence analogous to the problem of responding only to combinations of sensory features that uniquely identify objects [26]; conversely, the dFBP is the dual of the FBP (for a definition of duality see [34]); finally the pFBP is another form of the feature binding problem where the neuron fires for objects with overlapping features. We return to the general implications of identifying these binding problem functions in the Discussion.

Propositions 1 and 2 demonstrate that a Boolean function can be implemented using at least two different methods. These methods are either based on the disjunctive (DNF) or the conjunctive (CNF) normal form expressions. The DNF expression decomposes a Boolean function into a disjunction of terms: using this method each dendritic sub-unit computes a term, and an input vector elicits a somatic spike when this input vector activates at least one dendritic sub-unit. The CNF expression decomposes the Boolean function into a conjunction of clauses: using this method each dendritic sub-unit computes a clause, and an input vector elicits a somatic spike when this input vector activates all dendritic sub-units at the same time.

The DNF and CNF expressions provide rigorous methods for implementing Boolean functions where dendritic sub-units respectively correspond to terms or clauses. They are also respective examples of two distinct classes of implementation strategies: local strategies in which the activation of a single dendritic sub-unit can make the neuron spike for at least one input vector (e.g. the DNF-based method); and global strategies in the which a single dendritic sub-unit is insufficient to make the neuron spike for any input vector (e.g. the CNF-based method). Local and global correspond to two distinct categories of implementation method, the DNF-based or CNF-based methods are two extreme examples of these categories. Notably, when there is more than one dendritic sub-unit, we can distinguish multiple forms of local strategy according to how many of the N sub-units are able individually to make the neuron spike at least once (see Figure S4 in Text S1).

Our numerical analyses provide further examples of how this local vs global distinction can be observed even with only one non-linear dendritic sub-unit.

#### Both local and global strategies are possible with spiking dendrites

Proposition 1 shows that a sufficient number of spiking dendritic sub-units allows any Boolean function to be implemented using the DNF expression (e.g. a local implementation strategy). Moreover, it follows from the proposition 2 that a two-stage neuron with a sufficient number of spiking sub-units can also implement any Boolean function using the CNF expression (e.g. a global implementation strategy). Such dual construction is possible because a spiking dendritic sub-unit has both sub-linear and supra-linear components in its activation function (see Materials and Methods spiking and saturating dendritic activation functions). On the one hand, the supra-linearity ensures the possibity of a DNF implementation. On the other hand, the sub-linear part of the dendritic non-linearity ensures the possibility of a CNF implementation (as already pointed out in the proof of proposition 2). Thus, spiking sub-units allow, in principle, the implementation of any positive Boolean function using either a DNF or CNF based method. In other words, both local and global strategies are possible with spiking dendritic sub-units.

**Figure 3:**
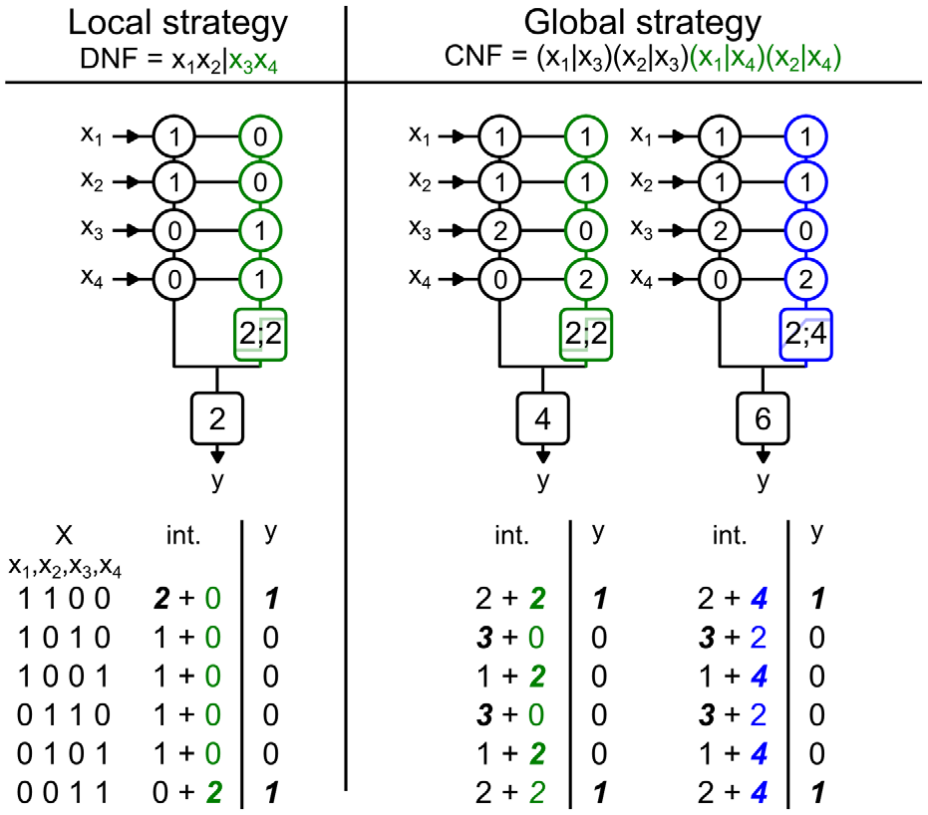
Two strategies to implement a linearly non-separable function. On top, the name of two possible strategies to implement the feature binding problem (FBP) based either on its DNF or CNF expression: the colored part of these expressions is the term or the clauses implemented by the dendritic sub-unit. Below, three schematics which represent parameter sets implementing FBP using either a spiking (green) or a saturating (blue) dendritic sub-unit. In circles are the value of synaptic weights (Black∶linear, green∶spiking, blue∶saturating); in colored squares (green∶spiking; blue∶saturating) are the parameters of the dendritic activation function [threshold;height], in black squares is the threshold 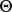 of the somatic sub-unit. Left, the local implementation strategy; Right, the global implementation strategy; note that a neuron cannot implement the FBP using the local strategy with a saturating dendritic sub-unit. Bottom, truth tables where the 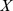 column is the input vectors, 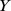 columns describe the neuron's input-output function, here the FBP. The int. column is the result of synaptic integration of each dendritic sub-unit (black∶linear, green∶spiking, blue∶saturating). In bold and italic are the maximum possible outputs for each sub-unit, note that for the global strategy a maximal output from a dendritic sub-unit may not trigger a somatic spike.

Our numerical analysis found multiple parameter sets that could implement the three functions using either a local or a global strategy. Figure 3 (left) shows an example of how a neuron with a spiking dendritic sub-unit can compute the FBP function using a local strategy. In this strategy the neuron's output is triggered either because of the direct stimulation of the somatic sub-unit (to input vector 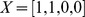), or because of a dendritic spike produced by the dendritic sub-unit (to input vector 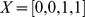); in both cases the other sub-unit contributed nothing to the whole neuron's output. Figure 3 (right) shows an example of how a neuron with a spiking sub-unit can compute the FBP function using a global strategy. In this case an input vector triggers an output spike when both the somatic and dendritic sub-units are stimulated simultaneously. Examples of a neuron with a single spiking dendritic sub-unit implementing the dFBP and pFBP using either a local or a global strategy are shown respectively in Figure S1 and Figure S2 in Text S1. Thus a neuron with a single additional spiking dendritic sub-unit can solve binding problems using either a local or a global implementation strategy.

#### A local strategy is impossible with saturating dendrites

We did not find parameter sets with a saturating dendritic non-linearity implementing a binding problem using a local strategy. The lack of parameter sets using this strategy was not a product of the parameter search, but rather a strict limitation of 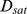. The informal proof by contradiction for the FBP is presented in Material and Methods.

We confirmed the generality of this result using the generic two-stage neuron model (Eq 2):

*Proposition 3*. A two stage neuron with non-negative synaptic weights and only dendritic units with saturating activation functions cannot implement a positive Boolean function based on its complete DNF

*Proof*. The activation function of a saturating dendritic unit is strictly sub-linear, therefore this unit cannot implement a term (Lemma 3).

This shows that irrespective of the number of saturating sub-units, the two-stage neuron model cannot compute Boolean functions using a local implementation strategy.

Nevertheless, a neuron with a saturating dendritic non-linearity (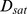) can compute the FBPs using a global strategy (Figure 3 and Figure S1, S2 in Text S1). Similar to a neuron with a spiking dendritic sub-unit, for this implementation strategy an input vector triggered the neuron output only if both the somatic and dendritic sub-unit are activated simultaneously.

These results suggest that spiking dendritic sub-units are more flexible than saturating dendritic sub-units. In principle, dendritic spikes allow the computation of Boolean functions through either a DNF (local) or CNF-based (global) method, whereas dendritic saturation cannot implement Boolean functions using a DNF-based (local) method. For certain Boolean functions, the length of the DNF and CNF differ significantly [39], implying a significant difference in the necessary range of synaptic weights to implement those functions [40]. A neuron with spiking sub-units can implement either the CNF or DNF decomposition, so it can be configured to use the smallest range of synaptic weights, whereas a neuron with saturating sub-units is restricted to the CNF decomposition. Consequently, for a neuron with a fixed, finite range of synaptic weights, it is probable that there exists a sub-set of Boolean functions inaccessible to neuron with only saturating dendrites and yet accessible with spiking dendrites. We showed previously [41] that the same limitation applies when the number of nonlinear dendritic sub-units is finite: in that study we provided an example of computation that might require an exponential number of saturating sub-units but requires a linear number of spiking sub-units.

### Linearly non-separable functions can be implemented in reduced and in realistic biophysical models

The preceding work used the framework of binary neuron models to establish the qualitative and quantitative gain in computational capacity obtained through the addition of a single dendritic non-linearity. This framework enabled us to study in detail how a linearly non-separable function can be implemented. Furthermore, this Boolean framework also enabled us to formally prove the results (Proposition 1,2,3) extracted from our large parameter searches (Figure 2). To demonstrate that this approach also gave us meaningful insights into biological single neuron computations, we implemented linearly non-separable Boolean functions in reduced and in realistic biophysical models; specifically, we showed that both the saturating and spiking forms of nonlinear dendritic summation enabled a neuron to implement linearly non-separable function in a more realistic framework.

#### Implementation of the FBP in reduced biophysical models

**Figure 4:**
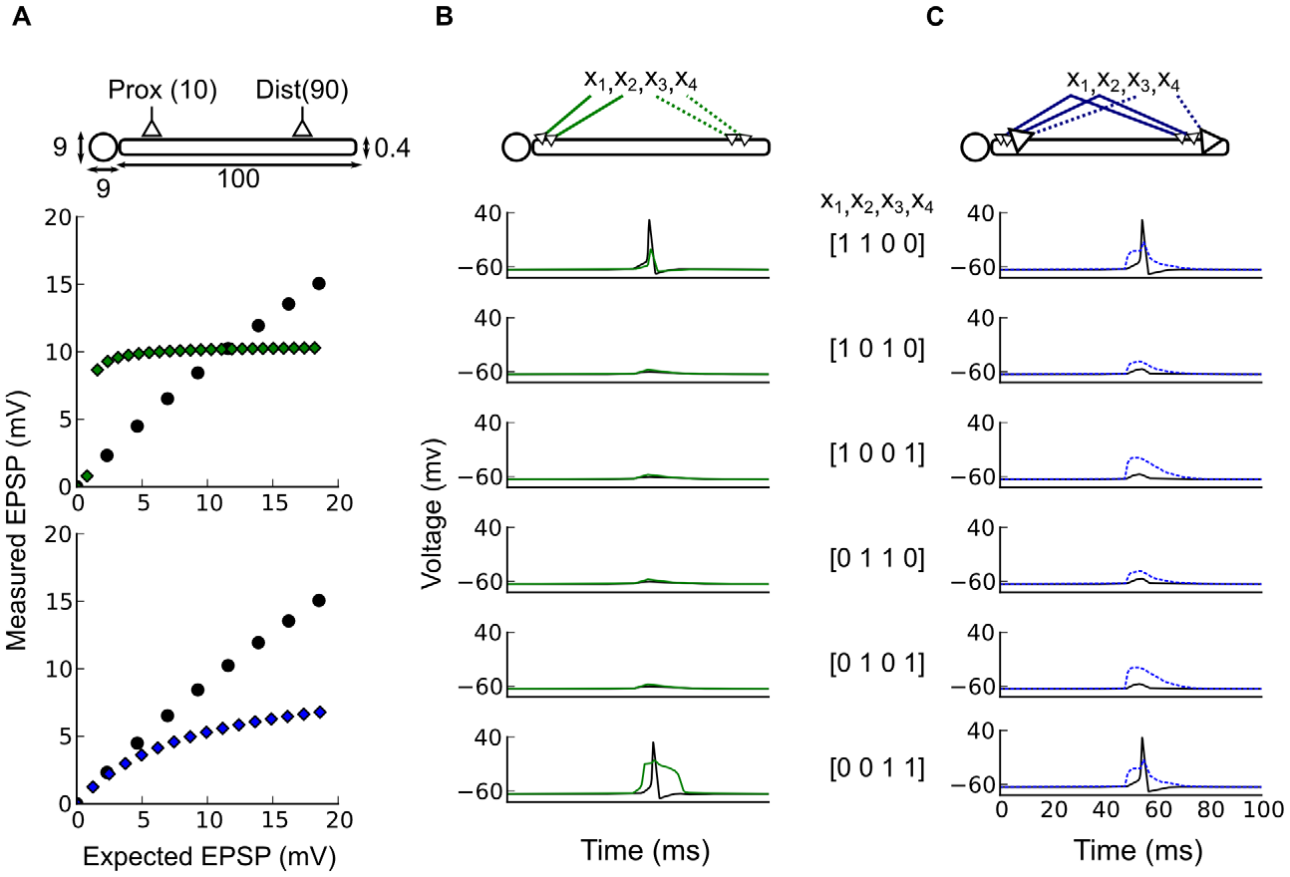
Reduced biophysical models can implement linearly non-separable functions using dendritic saturations or dendritic spikes. (A) The biophysical model. Upper panel: schematic representation of the biophysical model, where synaptic inputs are clustered (Prox and Dist), and its morphological parameters (input locations, diameters, and dendritic length are in 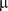
m). Below, expected arithmetic sum versus measured somatic EPSPs: for peri-somatic AMPA stimulation (black dots) producing a linear EPSP integration; for distal NMDA stimulation (green dots) producing a spiking type non-linear summation; for distal AMPA stimulation (in blue) producing a saturation type non-linear summation. (B) Implementation of the feature binding problem using NMDA receptor synapses for the distal dendritic region, illustrating the DNF/local strategy of synaptic placement. Top panel shows how each input makes synaptic contacts in a 10 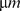 zone either on the peri-somatic or on the distal dendritic region. Below, voltage traces (black∶soma; green∶distal dendrites) in response to the various input patterns. Each voltage trace corresponds to stimulation by a different input vector where an active input variable is a neural ensemble of 100 neurons firing nearly synchronously in a 10 ms window. (C) Implementation of the feature binding problem using only AMPA synapses corresponding to a saturation type non-linear sub-unit and a CNF/global strategy. Top panel shows how each input makes synaptic contacts in a 10 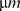 zone either on the peri-somatic or on the distal dendritic region. Below: voltage traces in response to the various input vectors (black∶soma; blue∶distal dendrites).

We implemented the FBP function in two-compartment Hodgkin-Huxley biophysical models containing a somatic and a dendritic compartment, as shown in Figure 4. The biophysical models provided a test of two assumptions made in our binary neuron model. First, we assumed that the sub-units summed their inputs independently, but in a real neuron the different sections of the neuron are electrically coupled. Here, a high intracellular resistance, 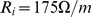 and a small dendritic diameter, 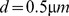 guaranteed a sufficient independence of the integration sites as discussed in [10]. Second, in the binary model we used discontinuous or peaked activation functions to model non-linear summation, but in neurons non-linear summation is a smooth function of the inputs. Consequently, in the biophysical model we used time-dependent AMPA conductances to generate saturations as shown on Figure 4A similar to the experimental result presented in Figure 1B. We also used NMDA time-dependent and voltage-gated conductances to generate dendritic spikes as shown on Figure 4A similar to the experimental result presented in Figure 1A. Consequently, our proof of principle model demonstrated not only how a neuron might implement linearly non-separable Boolean functions, but also how it can do so when moderately relaxing two assumptions: complete independence of sub-units and sharp activation functions.

Whereas EPSP summation in the peri-somatic region was linear, EPSP summation in the distal dendritic region was of either type of non-linearity, as shown on Figure 4A. The dendritic-spike nonlinearity was created by including only NMDA receptors in the dendritic distal dendritic region: their voltage-dependent depolarization of the membrane led to a step-like total EPSP response to multiple synaptic inputs, consistent with the measurements of Polsky et al [13]. The dendritic saturating non-linearity was a straightforward consequence of using conductance-based synaptic inputs: because the diameter of the distal dendrites was small, local depolarization due to a synaptic input was high, markedly reducing the local driving force 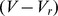; consequently, summation of multiple EPSPs was strongly sub-linear, consistent with the measurements of Abrahamsson et al [10].

To show that the biophysical model could compute the FBP function, we needed to define the mapping between the binary model and the biophysical model and what we meant by input and output. For the input, we considered here that the four input variables 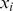 corresponded to four afferent neural ensembles of 100 neurons each, and that an ensemble was active, signaling 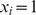, when all neurons in the ensemble fired a spike almost synchronously. For example, the input vector 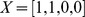 corresponded to two simultaneously active neural ensembles (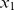 and 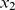) and the arrival of 200 pre-synaptic spikes in a 10 ms time window. For the output, we considered a single post-synaptic spike as the response to an input vector, such that if the neuron fired a somatic spike then this was equivalent to 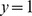, and no somatic spike was equivalent to 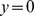. Thus, to implement the FBP function successfully, the biophysical model should only spike in response to the input vectors 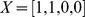 and 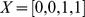, and not to any other of the six tested input vectors (corresponding to the truth table displayed in Table 2).

We used the results obtained previously with the binary model to set the weight and placement of the different synapses from each neural ensemble. Maximal conductances for each synapses ensemble were constrained to remain in the ratios of the weights found in binary neuron implementations in Figure 3. Similarly, placement of input ensemble synapses followed those example implementations, and thus inputs to a sub-unit were omitted if the weight was zero. With the conductances constrained to those ratios and placements, we easily found by hand values for which the biophysical neuron model implements the FBP function.

Figure 4B shows the successful implementation of the FBP function for the biophysical dendritic-spike non-linearity. For this model, we placed the inputs on the peri-somatic region and the distal dendrite following a local implementation strategy, meaning that each input stream targeted a single region, as shown in Figure 3 and Figure 4B. Thus, either a somatic spike was triggered directly, or the dendritic spike could in turn trigger a somatic spike, but any other combination of inputs correctly resulted in the absence of a somatic spike. Figure 4C shows the successful implementation of the FBP function by the biophysical model with a saturation type non-linearity. For this model, we placed the inputs on the peri-somatic and the distal dendrite following a global implementation strategy, meaning that each input stream targeted both regions, as shown in Figure 3 and Figure 4C. Thus, for this biophysical model, correctly triggering a somatic spike required sufficient stimulation of both the peri-somatic and the distal dendritic region. If only the peri-somatic or the distal dendritic region receives a strong stimulation, then the dendritic saturation correctly prevented a somatic spike.

#### Implementation of the dFBP in a cerebellar stellate cell

**Figure 5:**
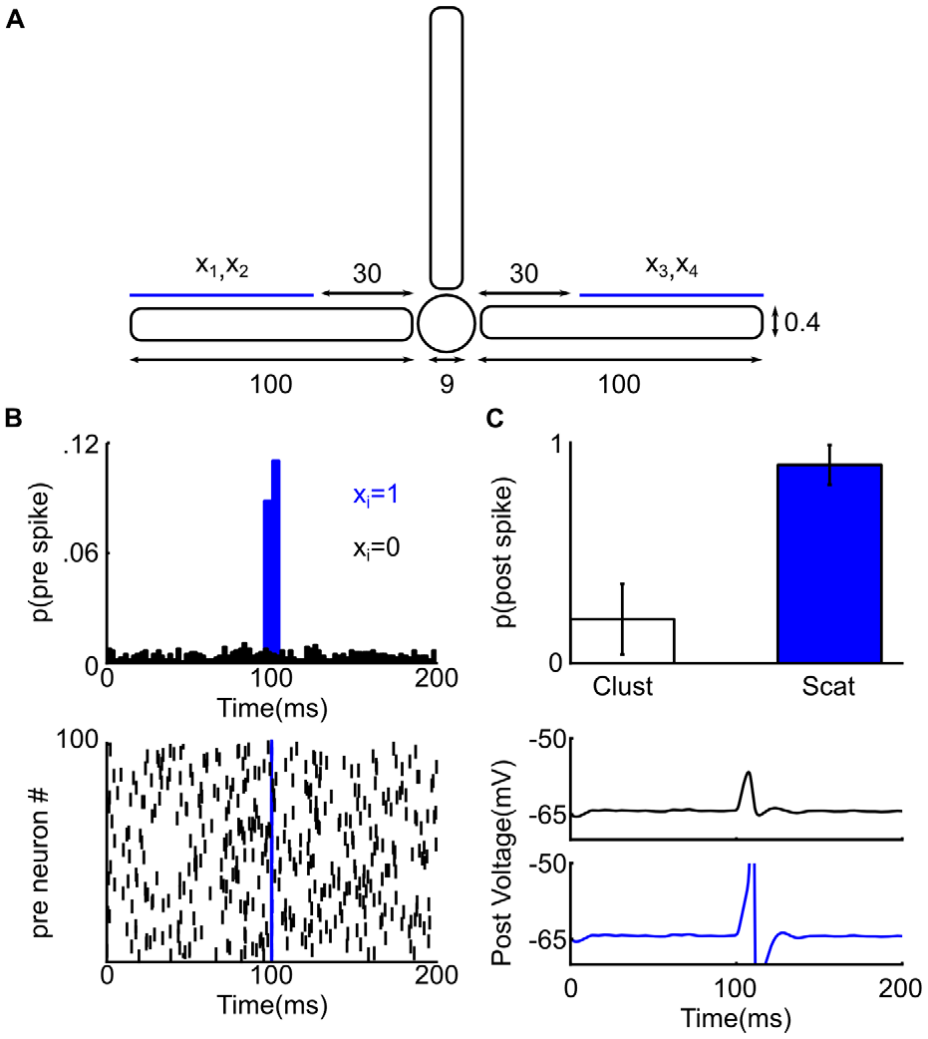
Cerebellar stellate cell interneurons can implement the dual feature binding problem. (A) A schematic representation of the biophysical model, the circle represents the soma and the 3 cylinders correspond to dendrites, their size is expressed in 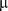
m, the blue bars represent the region where the four cell assemblies 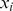, each made of 100 pre-synaptic neurons, makes contacts every 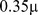
m, between 30 and 100 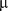
m away from the soma. 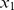 and 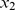 make contact on the the left dendrite, whereas 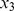 and 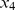 make contact on the right dendrite. (B) Above, a spike density plot of the cell assembly 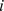, each made of 100 pre-synaptic neurons, when 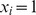 (blue) or 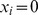 (black). Below, the corresponding raster plot. (C) Above, the probability of a post-synaptic spike averaged over 10 trials, when two scattered inputs are active (Scat: 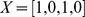, 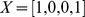, 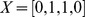, or 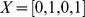) or when two clustered inputs are active (Clust: 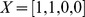 or 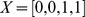). The bars correspond to the variance of the binomial distribution for p(post spike). Below, somatic voltage traces in clustered (black) or in scattered (blue) condition. Note that our model of cerebellar stellate cells fires significantly more often (Binomial test, 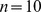 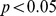) when inputs are scattered over the dendritic tree.

Next, we looked at how a realistic biophysical model can implement a linearly non-separable positive Boolean function. Figure 5 shows how the dFBP can be implemented in a realistic model of cerebellar stellate cell. This cell type is interesting because it is an experimentally-studied example of an electrotonically compact neuron with ‘passive’ dendrites [10]. Moreover, the same study demonstrated using patch-clamp and simultaneous glutamate uncaging that integration is strictly sub-linear in the distal dendritic region (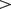
30 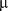
m away from the soma). These features make the cerebellar stellate cell an ideal candidate to see if our binary model leads to testable experimental predictions.

We used a global strategy with three non-linear passive dendrites to implement a linearly non-separable function. The four input streams 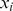, each made of 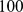 neurons, make synaptic contacts on the distal dendrites between 30 and 100 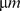 from the soma every 0.35 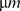. We split the input streams into two sets: (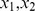) made synaptic contacts on one dendrite, (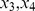) targeted another dendrite, and the third dendrite was not targeted. This synaptic placement corresponded to the implementation of the dFBP using a global strategy, as shown on Figure S1 in Text S1 and Figure 5A.

To test if a cerebellar stellate cell can actually implement the dFBP, we measured its membrane voltage trace in response to two different conditions. The scattered condition corresponded to the following input vectors, where inputs arrive at both targeted dendrites: 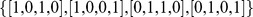 (which are equivalent due to symmetry). The clustered condition corresponded to the following input vectors, where inputs arrive at just one dendrite: 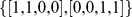 (which are again equivalent due to symmetry). In both conditions, the neuron received exactly the same number of spikes, so that the only difference was the spatial repartition of synaptic activation and not its magnitude. However, the probability of spiking for the cerebellar stellate cell model was significantly higher in the scattered condition than in the clustered condition (Binomial test with 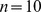 trials and 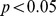), as illustrated on Figure 5C. This differential response to the scattered and clustered conditions revealed that our model of cerebellar stellate cell was successfully implementing an approximation to the dFBP.

Together, these biophysical models showed a proof-of-principle that a neuron with passive and compact dendrites can compute linearly non-separable Boolean functions.

## Discussion

Our study addressed the implication of non-linear dendritic summation of inputs for single neuron computation (for reviews see [42], [43], [44]). Previous theoretical studies which addressed this question assumed that two conditions may be necessary: (1) dendritic spikes [16] and (2) the existence of multiple non-linear sub-units [18]. These two conditions are true only for neurons that possess the right balance of voltage-gated channels in their dendrites to produce dendritic spikes (e.g. see [8]), and spatially extended dendritic trees to ensure sufficient electrotonic distance between dendritic sections (e.g. see [13]). In this paper we investigated whether these two conditions, dendritic spikes and a large number of independent sub-units, were necessary for neurons to compute linearly non-separable Boolean functions.

We have shown that a single non-linear dendritic sub-unit, spiking or not, is sufficient to increase a neuron's computational capacity. Using large parameter searches, we showed that a spiking or a saturating dendritic sub-unit enables a neuron to compute linearly non-separable functions. Moreover, we analytically proved that with a sufficient number of saturating dendritic sub-units a neuron could compute all positive Boolean functions (Proposition 2). To our knowledge, this is the first demonstration that passive dendrites can expand the number of Boolean function computable by a single neuron.

We have shown that a neuron with multiple dendritic sub-units can implement a Boolean function using either a DNF/local strategy, where each dendritic sub-unit can independently trigger the post-synaptic spike response, or a CNF/global strategy, where dendritic sub-units must cooperate to trigger a post-synaptic spike response. In the latter implementation strategy dendritic tuning does not imply the neuron tuning: a given stimulus can elicit the maximal response for a dendritic branch and yet elicit no response for the neuron, as observed in [35]. Moreover, we showed that a neuron with saturating dendritic sub-units cannot implement Boolean functions using a DNF/local strategy.

Finally, we used reduced and realistic biophysical neuron models to provide a proof-of-concept. We showed that a realistic model of cerebellar stellate cell interneuron can implement a linearly non-separable Boolean function.

### Sufficient conditions for changing the computational capacity of the single neuron

Our study has established three sufficient conditions for how nonlinear summation in dendrites increases the computational capacity of a single neuron. First, we showed that a single non-linear dendritic sub-unit is sufficient for a neuron to compute a significant amount of linearly non-separable functions. This result apparently differs from previous studies which found that a single non-linear sub-unit was insufficient to increase the capacity of neurons to compute arithmetic functions [18]. This previous study considered only dendritic non-linearities, but we have shown here that a dendritic non-linearity associated with the somatic non-linearity enables the computation of linearly non-separable functions. We have presented in the present study a situation where a spiking dendritic sub-unit did not trigger a somatic spike, as observed in [45], [46], [3], and still this sub-unit enabled the computation of a linearly non-separable function. Therefore a single dendritic branch is sufficient to boost the computational capacity of a neuron even if its non-linear region cannot trigger on its own a somatic spike. The existence of linear and non-linear integrating regions in our reduced models is supported by the recent demonstration that cerebellar stellate cells or layer 2/3 cortical pyramidal neurons can display both types of integration, even on single dendritic branches: linear EPSP summation in proximal/peri-somatic regions and nonlinear summation in distal regions [10], [36]. While this proximal-distal arrangement could amplify the distal EPSPs and compensate for the electrotonic distance between the inputs and the soma, as the authors argued in [47], [48], we showed how this arrangement can allow neurons to compute linearly non-separable functions.

Second, we showed that a saturating non-linear sub-unit is sufficient for a neuron to compute linearly non-separable functions. To the best of our knowledge, only a single prior study proposed that saturation can enhance single neuron computation, within the context of the coincidence detection in the auditory system [49]. Our study extends this result and show that a neuron with passive dendrites can compute linearly non-separable functions.

Third, we found that scattered synaptic contact is sufficient to implement linearly non-separable functions. This is in contrast to a prior study [26] which suggested that, to implement feature object binding problems, synaptic inputs carrying information about an object should cluster on a single dendritic sub-unit. We have shown here implementation strategies solving FBPs where inputs coding for separate features of the same object are distributed over the whole dendritic tree (see Figure 3 and Supplementary Figures S1 and S2 in Text S1).

### Linearly non-separable functions and Object-Feature binding problems

The positive linearly non-separable functions that a neuron can compute using a nonlinear dendritic integration can be described as feature binding problems (see Table 2) as defined in [26]: the task of signaling that separate elementary features belong to the same object, and are separate from features defining other objects. For example, a feature can be a sensory input corresponding to a position or an orientation of a bar and an object can be any conjunction of these two features, such as an oriented bar at a given location. The binding problem to be solved by the neuron is then to fire for a bar with a specific orientation present at a specific location, indicating its preferred object, and not to fire for any other combination of such features.

Binocular disparity [22] is a type of FBP problem: in this case input variables 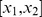 code for features coming from the left eye, and 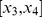 code for features coming from the right eye; the neuron then maximally responds if inputs only come from a single eye. Second, the generation of multiple place fields in single dentate gyrus cells [25] is a FBP problem; in this case 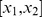 and 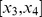 are independent sets of input features defining separate spatial locations, and the neuron maximally responds for either of these sets. Finally, binaural coincidence detection [49] is a dFBP problem; in this case 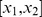 code for inputs coming from the left ear and 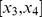 codes for inputs from the right ear; the neuron maximally responds if inputs come from both ears simultaneously. These example applications illustrate the ubiquity of FBP and dFBP-like problems solved by the brain, and consequently why implementations of these Boolean functions by single neurons may be important for understanding neural computation.

### Neuron vs dendrite as fundamental units of computation

The existence of non-linear summation in separate regions of the same neuron's dendrite suggests that a dendritic branch is a finer-grained computational unit than the whole neuron [50]. Our results show that, even when single neuron computation is enhanced by dendritic nonlinearities, it does not necessarily follow that there is a unit of computation smaller than the neuron. We found that a DNF/local strategy implementing such strict independence of dendritic sub-unit computation was possible, but only for the dendritic-spike nonlinearity. We found that an increase in computational capacity could be equally achieved by a global coordination of dendritic sub-units whether the dendritic nonlinearity was of saturating or spiking form. For such a global strategy, there is no sense in which each dendritic sub-unit separately computes a response to its inputs, and thus do not form an independent computational part of the whole function. A specific consequence of the CNF/global strategy is that separate dendritic regions have a different tuning from the whole neuron. This is consistent with Jia et al's [35] observation that a cortical layer 2/3 neuron can maximally respond to a given direction of moving gratings, even though individual dendritic branches are tuned to different orientations or to no orientation. Our results thus show how such a lack of evidence for independent dendritic integration does not imply a lack of dendritic computation. Therefore, the CNF/global implementation strategy suggests that non-linear dendrites may not replace neurons as a basic computational unit but rather expand neurons' computational capacities.

## Materials and Methods

### Spiking and saturating dendritic activation functions

A activation function 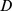 takes as input a local weighted linear sum 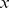 and output 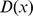, this output depends on the type of activation function: spiking or saturating, and on two parameters 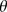, the threshold of the activation function, and 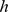, the height. The two type of activation functions are defined as follows:

*Spiking activation function*.
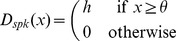



*Saturating activation function*.
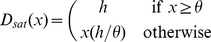
The difference between a spiking and a saturating activation functions is that 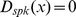 whereas 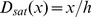 if 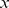 below 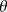. To formally characterize this difference we define here sub-linearity and supra-linearity of an activation function 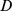 on a given interval 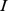. These definitions are analogous to the one given in [20]:

*Supra-linearity and sub-linearity*.

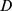
 is supra-linear on 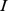 if and only if 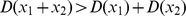 for at least one 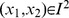

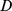
 is sub-linear on 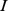 if and only if 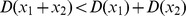 for at least one 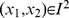

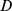
 is strictly sub-linear (resp. supra-linear) on 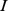 if it is sub-linear (resp. supra-linear) but not supra-linear (resp. sub-linear) on 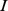.

Note that these definitions also work when using 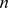-tuples instead of couples on the interval (useful in Lemma 3). Note that whatever 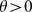, 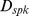 is both supra and sub-linear on 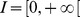 whereas 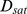 is strictly sub-linear on the same interval.


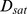
 is not supra-linear on 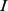 because 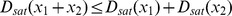 for all 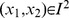, by definition of 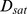. Moreover, 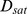 is sub-linear on 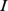 because 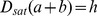 and 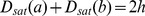 for at least one 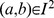 such that 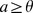 and 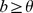. All in all, 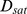 is strictly sub-linear on 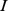.

Similarly to 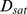, 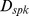 is sub-linear on 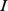 because 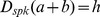 and 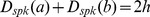 for at least one 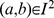 such that 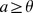 and 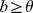. Moreover, 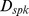 is supra-linear because 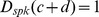 and 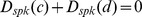 for at least one 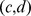 such that 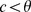 and 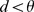 but 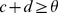. All in all, 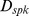 is both sub-linear and supra-linear.

Note that Maass [51] determined the upper limit on the computational capacity of networks made of piece-wise linear threshold functions. However, these activation functions are defined on 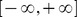 whereas a saturating activation function is defined on 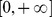, moreover, the ‘simplest’ studied examples of these type of activation functions are both sub-linear and supra-linear whereas a saturating activation function is strictly sub-linear.

### Boolean algebra

The input output mapping of a binary neuron model is a Boolean function. Let us recall some definitions of this extensively studied mathematical object [34], [52]:

**Boolean function.** A Boolean function 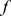 of 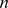 variables is a function on 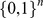 into 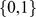, where 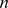 is a positive integer.

We first introduce a definition what will be useful for our numerical analysis:

**Representative Boolean function.** 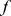 is representative of a set of functions 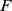 if 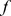 can generate all functions 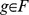 by permuting the labels of the input variables.

For example, for 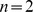, input vectors can be ordered as follows: 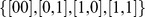; given these input vectors, the output vectors 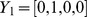 and 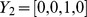 are two instances of a function set, as swapping the input labels such that 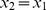 and 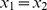 turns one into the other. This set can be represented by either 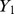 or 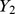. We define the computational capacity of a neuron as the number of representative functions it can compute for a given 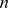.

Because of their importance here we recall the definition of positive Boolean functions and linearly separable Boolean functions:

**Positive functions.** Let 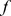 be a Boolean function on 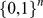. 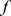 is positive 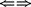 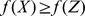 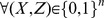 such that 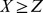 (meaning that 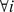 : 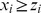)

**Linearly separable functions.** Let 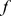 be a Boolean function on 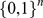. 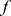 is linearly separable if and only if there exist a 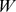 in 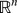 and a 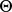 in 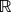 such that for all 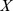


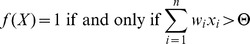
If there exist no such 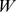 and 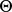, we said that 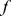 is linearly non-separable

In order to describe Boolean functions, it is useful to decompose them into positive **terms** and positive **clauses**:

**Terms and Clauses.**

- Let 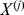 be a tuple of 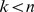 positive integers referencing the different variables present in a term or a clause.
- A positive term 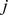 is a conjunction of variables written as 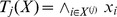.
- A positive clause 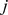 is a disjunction of variables written as 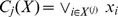.
- A term or (resp. clause) is prime if it is not implied by (resp. does not imply) any other term (resp. clause) in a disjunction (resp. conjunction) of multiple terms (resp. clauses).

These terms and clauses can then define the Disjunctive or Conjunctive Normal Form (DNF or CNF) expression of a Boolean function 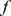, particularly:

**Disjunctive Normal Form (DNF).** A complete positive DNF is a disjunction of prime positive terms 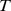:
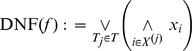



**Conjunctive Normal Form (CNF).** A complete positive CNF is a conjunction of prime positive clauses 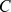:
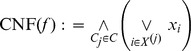
It has been shown that all positive Boolean functions can be expressed as a positive complete DNF ([34] Theorem 1.24); similarly all positive Boolean functions can be expressed as a positive complete CNF. These complete positive DNF or CNF are the shortest possible DNF or CNF descriptions of positive Boolean functions.

### Generation of representative positive Boolean functions

First, we generated the list of positive Boolean functions of 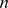 variables from the list of positive Boolean functions of 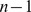 variables based on [53]. This method generates multiple times the same function, so we removed identical functions from the total list of positive functions.

Second, we extracted from this list of functions the set of representative Boolean functions. We sequentially enumerated the list of monotone Boolean functions; for each monotone function we permuted the input variables label to generate all its children. If none of these children were present in the list of representative functions - initially empty - we recorded the current monotone function in this list. Finally we checked whether the size of this list corresponded to the number of representative positive Boolean functions, which is equal to the number of NP-equivalence classes of unate functions of 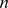 or fewer variables [37]. With our procedure we also found this number for 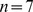, which is 490,013,148.

### Systematic search for a given parameter range

In the three different conditions (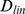, 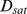, and 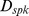), we systematically enumerated all the integer-valued sets of parameters for different parameter ranges up to the limits given in Table 1 for 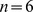. This Table displays the parameter values for which the number of computable Boolean functions stops growing. For instance for 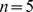 all positive linearly separable functions can be implemented in a linear model (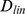) with integer-valued weights between 0 and 5, and a threshold between 0 and 9. For each parameter set we computed the associated Boolean functions; if this function was in the previously generated list of positive representative function we removed it from the list and recorded the set of parameters in a hdf5 data file. We then went to the next set of parameters and repeated the operation.

All these operations were programmed using python 2.7.1. We used numpy version 1.5 (www.numpy.org) for matrix operation, and h5py 1.3.1 (A. Collette, HDF5 for Python, 2008; http://h5py.alfven.org) to record the parameter sets in an hdf5 file.

This method provides lower bounds on the computational capacity of a neuron with a non-linear dendritic sub-unit. Therefore in 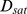 and 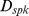 condition the actual computational capacity is superior to the one presented in Figure 2, because we may have missed parameter values for 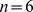.

### A neuron with saturating dendrites cannot implements the FBP using a local strategy

For the neuron to correctly produce output of 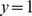 to both input vectors 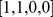 and 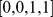 and for the strategy to be local means that:
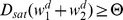
for response to 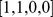, because the dendritic sub-unit triggers a somatic spike and
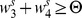
for response to 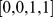, because in this case the dendritic sub-unit also triggers a somatic spike. In each case, one weight is necessarily larger than or equal to the other; let it be 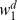 for the first equation and 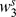 for the second. As 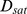 is strictly sub-linear (for definition see Materials and Methods), it follows that:
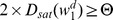
and
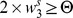
As each weight is thus at least 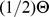, we can add these two inequalities to obtain:
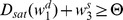
Finally, as adding any positive weight to the left-hand side does not change the sign of the inequality, so the neuron must also output 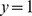 for input 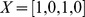, giving a false positive. Therefore the FBP function cannot be computed using a saturating nonlinearity and a local implementation strategy.

### Lemmas used to prove our propositions

**Lemma 1.** A two stage neuron with non-negative synaptic weights and increasing activation functions necessarily implements positive Boolean functions

**Proof.** Let 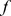 be the Boolean function representing the input-output mapping of a two stage neuron, and two binary vectors 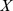 and 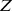 such that 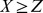. We have 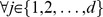 non-negative local weights 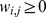, so for a given dendritic unit 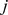 we have:
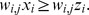
We can sum inequalities for all 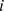, and because 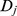 are increasing activation functions:
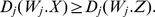
We can sum the 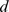 inequalities corresponding to every dendritic unit. As 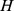, the Heaviside activation function of the somatic unit is increasing we obtain:
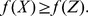



**Lemma 2.** A term (resp. a clause) can be implemented by a unit with a supra-linear (resp. sub-linear) activation function

**Proof.** We need to provide the parameter sets of a activation function implementing a term (resp. a clause) with the constraint that the activation function is supra-linear (resp. sub-linear). Indeed, a supra-linear activation function (like the spiking activation function) with the parameter set 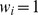 if 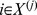 and 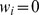 otherwise and 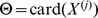 implements the term 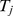. A sub-linear activation function (like the saturating activation function) with the parameter set 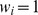 if 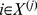 and 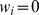 otherwise and 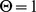 implements the clause 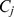.

**Lemma 3.** A term (resp. a clause) cannot be implemented by a unit with a strictly sub-linear (resp. supra-linear) activation function

**Proof.** We prove this lemma for a term, the proof is similar for a clause. Let 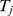 be the term defined by 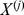, with 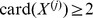. First, for all input vectors 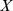 such that 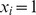 with 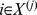 and 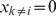 it follows that 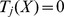, implying that 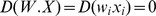. One can sum all these elements to obtain the following equality 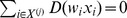. Second, for all input vectors 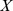 such that 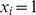 for all 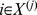 then 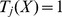 implying that 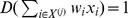. Putting the two pieces together we obtain:
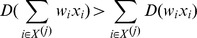
This inequality shows that the tuple of points 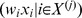 defining a term must have 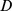 supra-linear. Therefore, by Definition 2, 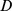 cannot be both strictly sub-linear and implement a term.

A formal treatment of the three propositions is also given in [41].

### Biophysical model

We built the compartmental biophysical models with NEURON software version 7.1 [54] coupled with Python 2.7.1. (www.python.org) Morphological parameters (e.g. dendrites' diameters) are described in the Figure 4A. The axon is not modeled because of its negligible contribution to the conductance load.

For the reduced models, the majority of parameters are set to their default value within this NEURON version. The active membrane parameters are the standard Hodgkin-Huxley channels (hh in NEURON) also used with their default parameters; the default and non-default parameters defining passive and active properties of this model are given in Table 3. To model AMPA synapses we used the built-in Exp2Syn synapses; for NMDA synapses we used nmdanet.mod from [55]. The range of tested synaptic weights which correspond to the maximum conductance and the synaptic parameters are described in Table 3. We used biophysical modeling to illustrate the mapping between our abstract binary neuron model and a full multi-compartment dynamical neuron model. We designed the scripts for testing the biophysical models input-output mapping such that they can be used with any type of arbitrarily detailed biophysical neuron model; the script is available in the ModelDB (when this manuscript will be accepted).

**Table 3:** The parameters for the biophysical models.

Parameters	Value
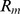	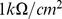
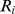	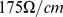 from [10]
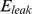	−65 mV
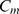	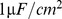
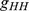 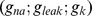	0.120 ; 0.0003 ; 0.036 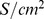
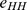 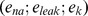	50 ; −77 ; −54.3 mV
AMPAR (reduced model)	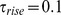 , 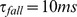
	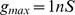 in total
AMPAR (realistic model)	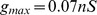 for each synapses
NMDA	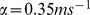 , 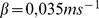
	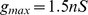 in total
Synaptic reversal potential( 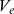 )	0 mV
Number of segments per section	3 for soma and 9 for dendrites

For the realistic model of cerebellar stellate cells, the morphological and biophysical parameters are taken from [10].

## Supporting Information

Text S1 Implementation of different feature binding problems. We describe in this supplementary text how the pFBP and dFBP can be implemented with a saturating or spiking dendritic subunit. We also demonstrate how the FBP and the dFBP can be implemented with a number of non-linear dendritic subunits linearly proportional to the number of objects. This supplementary text also shows how the pFBP can be implemented using an intermediate strategy between local and global ones.
(PDF)
