# Supplementary material for: Passive Dendrites Enable Single Neurons to Compute Linearly Non-separable Functions
Source: PLoS Comput Biol. 2013 Feb 28;9(2):e1002867. doi: 10.1371/journal.pcbi.1002867 (PMC3585427; doi:10.1371/journal.pcbi.1002867)
Supplement: Text S1 — Implementation of different feature binding problems. We describe in this supplementary text how the pFBP and dFBP can be implemented with a saturating or spiking dendritic subunit. We also demonstrate how the FBP and the dFBP can be implemented with a number of non-linear dendritic subunits linearly proportional to the number of objects. This supplementary text also shows how the pFBP can be implemented using an intermediate strategy between local and global ones. (PDF) [file pcbi.1002867.s001.pdf]

A spiking subunit is sufficient to solve FBPs and dFBPs for an arbitrary number of input variables

We demonstrated in the main text a computational distinction between spiking and saturating dendrites that a neuron with only saturating dendrites is incapable of using a local strategy. Is this distinction an important issue? Here we show that a neuron with a single spiking dendrite can compute the FBP with two objects and the corresponding dFBP for an arbitrary number of input variables n. Moreover, this computation is possible using binary synaptic weights instead of unbounded integer-valued synaptic weights. With binary weights an implementation is always possible in practice. By contrast, unbounded integer weights imply that each synapse should be capable of maintaining multiple distinct values, which may be impossible [1]. A neuron with a single saturating dendrite cannot compute the FBP for an arbitrary n with binary synaptic weights.

A neuron with a single spiking dendritic sub-unit can compute the $FBP = O_1|O_2$ for an arbitrary number of features composing the two objects O, i.e. $O_1 = x_1x_2\dots x_p$ and $O_2 = x_{p+1}x_{p+2}\dots x_q$. To solve this problem it is sufficient to add synaptic connections of weight 1 to the somatic non-linearity for features from O_1 and to the dendritic non-linearity for features from O_2, to set the dendritic threshold to $r = q - p$ with a height of p, and to set the somatic threshold to p, as shown in Figure S3A. By contrast, for $n = 4$ the implementation of the FBP with a saturating dendrite is impossible with binary weights. This is certain as our parameter searches were exhaustive for binary weights. It suggests that the implementation of the FBP for arbitrary n using a saturating dendrite and binary weights is impossible.

Yet a neuron with a saturating dendrite can compute the $dFBP = F_1F_2$ for arbitrary size of the two sets of independent features F, i.e. $F_1 = x_1|x_2|\dots|x_p$ and $F_2 = x_{p+1}|x_{p+2}|\dots|x_q$. To solve this problem it is sufficient to add synaptic connections of weight 1 to the somatic non-linearity for features from F_1 and to the dendritic non-linearity for features from F_2, to keep the dendritic threshold to 1 but set its height to p, and to set the somatic threshold to $p + 1$, as shown on Figure S3B. Note that the same implementation is possible with a spiking dendrite.

With more dendritic subunits N-Local implementations are possible

Local and global implementation strategies are a strict classification either a dendritic subunit can fire the neuron or no single sub-unit can fire the neuron. If there is more than one dendritic non-linear subunit then we can usefully distinguish different forms of local strategies. We say that an implementation is N-local when N dendritic subunits can fire the neuron. Figure 4 provides examples where the strategy is either 2-local, 1-local, or global (0-local). It shows that global and N-local implementations, e.g. DNF or CNF-based, are two extremes of a spectrum. There are many possible implementations in between these two extremes.

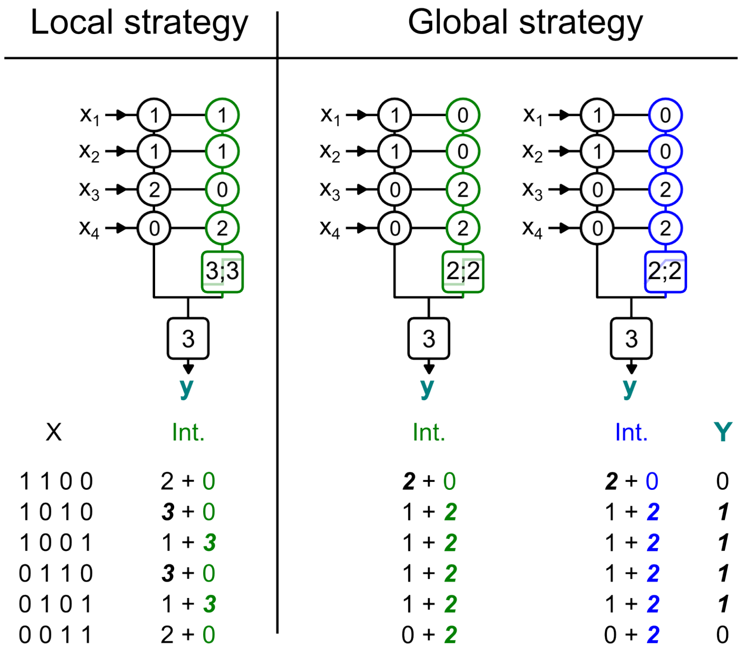

Figure S 1: Implementation of the dFBP using a local or global strategy. Above, three schematics which represent parameter sets implementing the dual feature binding problem (dFBP) using either a spiking (green) or a saturating (blue) dendritic sub-unit. In circles are the value of synaptic weights (Black:linear, green:spiking, blue:saturating); in colored squares (green:spiking; blue:saturating) are the parameters of the dendritic activation function [threshold;height], in black squares is the threshold Θ of the somatic sub-unit. Left, the local implementation strategy; Right, the global implementation strategy; note that a neuron cannot implement the FBP using the local strategy with a saturating dendritic sub-unit. Below, truth tables where the X column is the input vectors, Y columns describe the neuron's input-output function, here the FBP. The int. column is the result of synaptic integration of each dendritic sub-unit (black:linear, green:spiking, blue:saturating). In bold and italic are the maximum possible outputs for each sub-unit, note that for the global strategy a maximal output from a dendritic sub-unit may not trigger a somatic spike.

References

[1] Johanna M Montgomery and Daniel V Madison. Discrete synaptic states define a major mechanism of synapse plasticity. *Trends in neurosciences*, 27(12):744–50, December 2004.

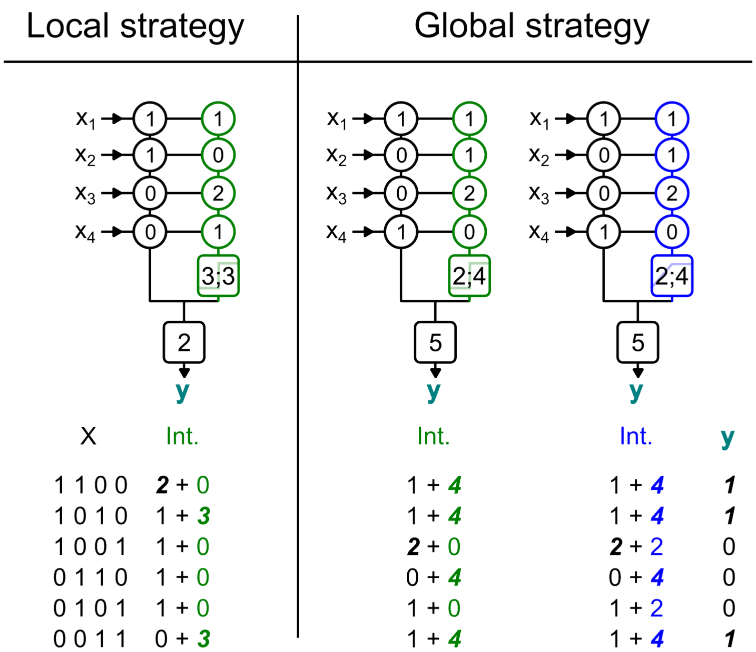

X	Int.	Int.	Int.	y
1 1 0 0	***2*** + 0	1 + ***4***	1 + ***4***	***1***
1 0 1 0	1 + ***3***	1 + ***4***	1 + ***4***	***1***
1 0 0 1	1 + 0	***2*** + 0	***2*** + 2	0
0 1 1 0	1 + 0	0 + ***4***	0 + ***4***	0
0 1 0 1	1 + 0	1 + 0	1 + 2	0
0 0 1 1	0 + ***3***	1 + ***4***	1 + ***4***	***1***

Figure S 2: Implementation of the pFBP using a local or global strategy. Above, three schematics which represent parameter sets implementing the partial feature binding problem (pFBP) using either a spiking (green) or a saturating (blue) dendritic sub-unit. In circles are the value of synaptic weights (Black:linear, green:spiking, blue:saturating); in colored squares (green:spiking; blue:saturating) are the parameters of the dendritic activation function [threshold;height], in black squares is the threshold Θ of the somatic sub-unit. Left, the local implementation strategy; Right, the global implementation strategy; note that a neuron cannot implement the FBP using the local strategy with a saturating dendritic sub-unit. Below, truth tables where the X column is the input vectors, Y columns describe the neuron's input-output function, here the FBP. The int. column is the result of synaptic integration of each dendritic sub-unit (black:linear, green:spiking, blue:saturating). In bold and italic are the maximum possible outputs for each sub-unit, note that for the global strategy a maximal output from a dendritic sub-unit may not trigger a somatic spike.

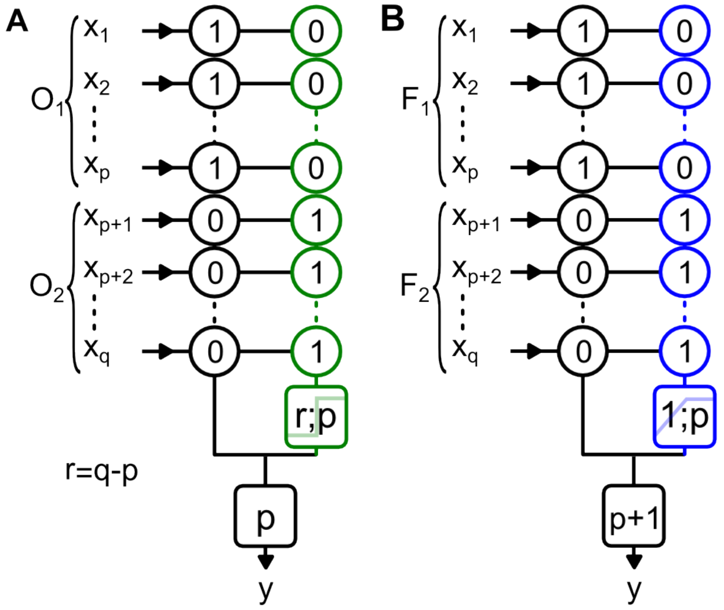

Figure S 3: Implementation of FBP and dFBP for an arbitrary n. Sets of synaptic weights are in the circles, the parameters of the dendritic non-linearity are in the colored squares (Threshold, Height), and the parameter of the somatic non-linearity is in the black squares. (A) Implementation of the FBP employing a spiking dendritic sub-unit, objects O are sets of features where each feature is represented by an x. (B) Implementation of the dFBP employing a saturating dendritic sub-unit, there are two sets of features F where each feature is represented by an x. We use for this implementation a global strategy therefore the dendritic sub-unit cannot make the neuron fire. The neuron spikes only when both sub-units are simultaneously stimulated. Note also that an implementation with a spiking dendritic sub-unit using the same parameter set is also possible.

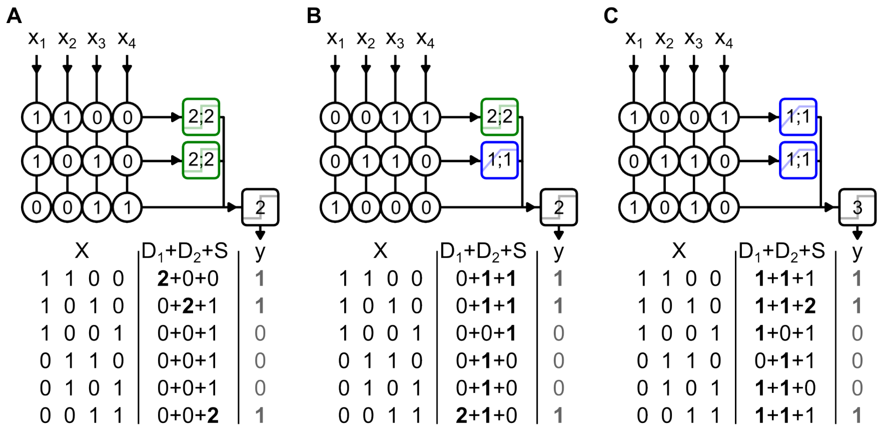

Figure S 4: Implementation of the pFBP employing two non-linear subunits. Sets of synaptic weights are in the circles, the parameters of the dendritic non-linearity are in the colored squares (Threshold, Height), and the parameter of the somatic non-linearity is in the black squares. (A) A 2-local implementation of pFBP where the two dendritic subunits can make the neuron spike, each subunit corresponds to a term. (B) A 1-local implementation where only one of the two dendritic subunits can make the neuron spike (C) A global or 0-local implementation where no dendritic subunit alone can fire the neuron; each subunit corresponds to a clause.
